# Potential effects of the combination of nicotinamide, vitamin B2 and vitamin C on oxidative-mediated hepatotoxicity induced by thioacetamide

**DOI:** 10.1186/s12944-018-0674-z

**Published:** 2018-02-14

**Authors:** Samir A. E. Bashandy, Hossam Ebaid, Sherif A. Abdelmottaleb Moussa, Ibrahim M. Alhazza, Iftekhar Hassan, Abdulaziz Alaamer, Jameel al Tamimi

**Affiliations:** 1 Pharmacology Department, Medical Division, National Research Centre, Bohouth St. (former EL Tahrir St.), Dokki, Giza, EL 33 Egypt; 2 Department of Zoology, College of Science, King Saud University, Riyadh, Kingdom of Saudi Arabia; 3 Department of Zoology, Faculty of Science, Minia University, Minia, Egypt; 4 Committee of Radiation and Environmental Pollution Protection (CREPP), Department of Physics, College of Science, Al- Imam Mohammad Ibn Saud Islamic University (IMSIU), Riyadh, Saudi Arabia; 5 Biophysics Group, Biochemistry Department, Genetic Engineering and Biotechnology Division, National Research Centre, Dokki, Giza, Egypt

**Keywords:** Hepatotoxicity, Thioacetamide, Nicotinamide, Vitamin B2, Vitamin C, Inflammatory markers, Lipid profile, Oxidative stress

## Abstract

**Background:**

The liver disease is one of the most important traditional public health problems in Egypt. Oxidative stress is attributed to such pathological condition that further contributes to the initiation and progression of liver injury. In the present study, we have investigated if the strong antioxidant power of Nicotinamide (NA), Vitamin B2 (VB2), and Vitamin C (VC) can ameliorate TAA-induced oxidative stress-mediated liver injury in the rats.

**Methods:**

Thirty-six albino rats were divided into six groups: Control group; TAA group (IP injection with TAA at a dosage of 200 mg/Kg three times a week for two months); TAA + NA group (rats administered with NA at a dosage of 200 mg/kg daily besides TAA as in the control); TAA + VB2 group (rats administered with vitamin B2 at a dosage of 30 mg/kg daily besides injection with TAA); TAA + VC group (rats administered with vitamin C at a dosage of 200 mg/kg daily along with injection of TAA). TAA + NA + VB + VC group (rats administered the with the three vitamins daily in TAA pre-injected at the respective doses described above).

**Results:**

Treatment of rats with TAA led to a significant elevation of aspartate aminotransferase (AST), alanine aminotransferase (ALT), alkaline phosphatase (ALP), lactate dehydrogenase (LDH), total bilirubin, cholesterol, triglycerides, low-density lipoprotein (LDL) and tumor necrosis factor-alpha (TNF-α) in the serum samples. Moreover, malondialdehyde (MDA), hydroxyproline and nitic oxide (NO) were also significantly increased in the TAA-treated rats, while reduced glutathione (GSH), superoxide dismutase (SOD) and catalase (CAT) were significantly compromised in the hepatic samples. Rats administered with NA, VB2, and VC as individually or in combination ameliorated the deleterious effects of TAA that was confirmed by histopathology. However, the combination of the three vitamins was found more effective as compared to each of the vitamins.

**Conclusion:**

Our work demonstrates that NA, VB2, and VC cross-talk with each other that act as a more potent biochemical chain of antioxidant defense against TAA-induced toxicities in vivo.

## Background

Egypt has been a hot spring of academic research under tropical medicine programs. Due to such medical problems, the confronting physicians have been facing the complications of hepatic fibrosis and cirrhosis because of schistosomiasis and chronic viral infections. It led many of the prominent hepatologists to establish clinical liver centers in entire Egypt region [1]. Four over three decades, many investigators have been trying to find efficient and long-lasting clinical solutions for liver diseases.

Thioacetamide (TAA) is a thio-sulfur-containing compound, which has been frequently used in leather, motor fuel, textile, paper, food, and beverage industries since long [2]. Administration of one dose of TAA leads to acute hepatic toxicity, while chronic exposure causes hepatic cirrhosis and possible development of liver tumors. The metabolic activation of TAA by cytochrome P450 generates TAA-intermediates, and reactive oxygen species (ROS) that can further covalently bind to biologically significant molecules and increase cellular oxidative stress, lipid peroxidation, and deplete glutathione [3]. Nicotinamide (NA), is a precursor of essential coenzymes for numerous reactions in the body [4]. Although NA is known to prevent or treat pellagra [5], it has other pharmacological actions as an antioxidant [6], and antinociceptive [7]. Nicotinamide, the amide derivative of vitamin B3, has been shown to exhibit a number of anti-inflammatory properties including inhibition of inducible NO synthase (iNOS) [8], free radical scavenging [9], suppression of MHC class II expression [10] and intracellular adhesion molecule ICAM-1 expression on endothelial cells [11]. NA is changed to nicotinamide adenine dinucleotide by the aid of enzymes. These cellular pathways are essential for energy metabolism and may directly influence normal physiology, as well as disease progression [12]. For example, NA has been shown to protect against bleomycin-induced Pulmonary Fibrosis [13], and doxorubicin-induced nephrotoxicity [14].

Riboflavin (VB2) is the central component of the cofactors Flavin adenine dinucleotide (FAD). VB2 plays a key role in cellular function, growth, development and energy metabolism [15]. It is effective in preventing migraine [16] and reduces hepatocellular injury following liver ischemia through its role in lowering hepatic inducible nitric oxide synthase and nitric oxide [17]. VB2 can act against oxidative stress, especially lipid peroxidation and oxidative injury. It is reported that riboflavin protects the body against oxidative stress by glutathione redox cycling beside conversion of the reduced riboflavin to the oxidized form among other possible mechanisms [18, 19]. Vitamin C (VC) is the most important radical scavenging antioxidant in extracellular fluids [20] Vitamin C (ascorbic acid) is a six-carbon lactone is called an antioxidant because, by donating its electrons, it prevents other compounds from being oxidized [21]. VC has been reported to attenuate hepatic damage and can act synergistically with other vitamins in the management of oxidative stress [22]. Vitamin C was reported to form a synergy with other agents to protect the liver. One of the obvious synergies is between vitamin C and vitamin E that ameliorated ethanol-induced hepatotoxicity via normalization of transaminases, lipid peroxidation [23] and endogenous antioxidants [24].

Thus, this study aimed to investigate hepatoprotective activities of nicotinamide, vitamin B2, and vitamin C, separately or in combination, against thioacetamide-induced liver damage, hyperlipidemia and oxidative stress in rats. The hepatoprotective activity was assessed using liver function tests, lipid profile, hepatic oxidative stress parameters, inflammatory markers and hydroxyproline as hepatic fibrosis marker.

## Methods

### Chemicals

TAA, NA, vitamin B2 and vitamin C were purchased from Sigma-Aldrich Corporation (St Louis, MO, USA). Remaining all chemicals were of analytical grade from authentic companies of International repute.

### Animals

The study was performed on six weeks’ old male albino rats weighing 120–150 g, obtained from the Egyptian Holding Company for Biological Products and Vaccines, Cairo, Egypt. The animals were kept in an environment with controlled temperature (25 °C), humidity (45–55%), and photoperiod (12-h/12-h light/dark cycle). All the animals under study had free access to chow diet and water. All animal care protocols were by the Research Ethics Committee, National Research Centre, Egypt.

### Experimental design

The rats were sorted into six groups, each containing eight rats as follows:

- Group I: Control rats without any treatment.
- Group II: Rats injected IP with TAA at a dosage of 200 mg/Kg [25] thrice a week for two months.
- Group III: Rats administered with NA at a dosage of 200 mg/kg [26] daily and injected with 200 mg/Kg of TAA IP thrice a week for two months.
- Group IV: Rats administered with vitamin B2 at a dosage of 30 mg/kg [27] daily and injected with 200 mg/Kg of TAA IP thrice a week for two months.
- Group V: Rats administered with vitamin C at a dosage of 200 mg/kg [28, 29] daily and injected with 200 mg/Kg of TAA IP thrice a week for two months.
- Group VI: Rats administered with all three of the previous vitamins daily and injected with 200 mg/Kg of TAA IP thrice a week for two months. The ratio of a multivitamin was performed as previously described [30].

### Samples collection

Blood samples were collected from each group by puncture of the retro-orbital venous sinus, into heparinized tubes under light ether anesthesia weekly. The blood was centrifuged at 1200×g for 10 min to separate plasma which was stored at − 40 °C. After collection of the blood samples, the animals from all the groups were autopsied under light ether anesthesia. The liver was removed from its surrounding tissues, placed into tubes and washed with normal saline (cold) and kept at − 40 °C. The liver tissues were homogenized in a cold potassium phosphate buffer (0.05 M, pH 7.4) and centrifuged at 3000 rpm for 10 min at 4 °C. The resulting supernatant of tissue homogenate was used for determination of the oxidative stress parameters.

### Biochemical and inflammatory parameters assay

Serum AST, ALT, ALP, LDH, total bilirubin, total protein, cholesterol, triglycerides, LDL and HDL concentrations were determined calorimetrically using the commercial kits (Salucea Company, Netherlands). Moreover, hepatic MDA, GSH, CAT, SOD and NO were determined calorimetrically using kits manufactured by Bio-diagnostic, Egypt. Plasma TNF-α and hepatic hydroxyproline were determined by enzyme-linked immunoassay by the kits from R&D Systems (USA) and Koma Biotechnology (Seoul, Korea) respectively.

### Histological studies

At the end of the experiment, a portion of a liver from each of the sacrificed rat was fixed in 10% buffered formalin and processed for paraffin sectioning after dehydrating them in various concentrations of alcohol, cleared with xylol and embedded in paraffin blocks. Sections of about 5 μm thickness were stained with hematoxylin and eosin (H&E) for histological study.

### Statistical analysis

Data have been presented as mean ± S.E.M and analyzed by one-way ANOVA followed by least significant difference (LSD) using SPSS software (version 20.0, SPSS Inc., Chicago, IL).

## Results

### Liver function tests

The mean values illustrated in Table 1 showed a significant elevation of plasma AST, ALT, ALP, LDH and total bilirubin in rats treated with TAA only, while total protein decreased compared to the control. These effects on liver function parameters associated with injection with TAA were alleviated when the rats were also treated with NA, VB2 or VC, with a significant decrease in the liver enzyme levels and bilirubin and an increase in total protein. Administration of all three vitamins together had a more profound effect in mitigating the adverse parameters than when the rats were treated with just one of the vitamins.

**Table 1:** Effect of Nicotinamide, Vitamin B2 and Vitamin C on liver function parameters in plasma of thioactamide treated rats

Treatment Parameter	Control	TAA	TAA + NA	TAA + VB2	TAA + VC	TAA + NA,VB2,VC
AST(U/l)	46.53 ± 1.08	269.36 ± 11.75^a^	137.64 ± 6.83^a,b^	142.43 ± 7.76^a,b^	170.34 ± 7.33^a,b^	95.17 ± 4.59^a,b,c^
ALT(U/l)	23.17 ± 1.34	153.46 ± 8.63^a^	114.56 ± 4.78^a,b^	109.89 ± 9.32^a,b^	94.57 ± 3.21^a,b^	65.46 ± 1.96^a,b,c^
ALP(U/l)	150.46 ± 10.06	676.35 ± 21.67^a^	470.48 ± 26.41^a,b^	418.34 ± 15.68^a,b^	580.41 ± 19.73^a,b^	242.00 ± 15.47^a,b,c^
LDH	212.55 ± 8.69	802.40 ± 4.75^a^	512.07 ± 18.67^a,b^	450.82 ± 14.25^a,b^	478.16 ± 17.56^a,b^	275.44 ± 12.49^a,b,c^
Total bilirubin(mg/dl)	0.43 ± 0.01	0.91 ± 0.03^a^	0.63 ± 0.01^a,b^	0.70 ± 0.04^a,b^	0.66 ± 0.03^a,b^	0.54 ± 0.02^a,b,c^
Total protein(g/dl)	7.34 ± 1.58	5.27 ± 0.10^a^	5.88 ± 0.19^a,b^	6.09 ± 0.27^a,b^	6.47 ± 0.34^a,b^	7.55 ± 0.10^a,b,c^

^a^Significant difference from control

^b^Significant difference from TAA group

^c^Significant difference from TAA + NA, TAA + VB2, TAA + VC

### Lipid profile

TAA injection resulted in a significant increase in the plasma level of cholesterol, triglycerides, and LDL, while HDL decreased significantly (Table 2). Treating the rats with TAA and NA, VB2 or VC, however, resulted in the changes in these parameters being reversed to some extent. Once again, a greater degree of improvement was noticed when the three vitamins were combined.

**Table 2:** Effect of Nicotinamide,Vitamin B2 and Vitamin C on plasma lipid profile of rats treated with thioacetamide

	Control	TAA	TAA + NA	TAA + VB2	TAA + VC	TAA + NA,VB2, VC
Cholesterol(mg/dl)	70.33 ± 3.45	158.58 ± 5.67^a^	135.11 ± 4.39^a,b^	114.37 ± 7.01^a,b^	102.44 ± 3.12^a,b^	84.37 ± 2.49^a,b,c^
Triglycerides (mg/dl)	64.58 ± 3.16	190.86 ± 8.23^a^	159.11 ± 4.23^a,b^	140.27 ± 3.55^a,b^	96.75 ± 2.81^a,b^	72.60 ± 5.36^b,c^
HDL (mg/dl)	33.62 ± 1.38	26.09 ± 1.43^a^	31.96 ± 1.02	30.25 ± 0.78	30.43 ± 1.54	32.48 ± 2.57
LDL(mg/dl)	49.72 ± 1.62	100.83 ± 3.58^a,b^	70.39 ± 3.08^a,b^	76.00 ± 2.51^a,b^	66.42 ± 2.81^a,b^	53.17 ± 1.94^b,c^

^a^Significant difference from control (P

^b^Significant difference from TAA group

^c^Significant difference from TAA + NA, TAA + VB2, TAA + VC

### Oxidative stress parameters

The data relating to hepatic oxidative stress markers are summarized in Table 3 and show a significant decrease in hepatic GSH, CAT, and SOD in the TAA group, but a marked increase in hepatic MDA, NO, Hydroxyproline and plasma TNF-α. These changes were significantly reversed in rats treated with the three vitamins in combination.

**Table 3:** Effect of Nicotinamide, Vitamin b2 and Vitamin C on hepatic MDA (nmol/mg), GSH (μmol/g tissue), catalase (U/g tissue), SOD **(U/g tissue),** NO (nmol/g,Hydroxyproline (Ug/g tissue) and plasma TNF-α (Pg/ml)

	Control	TAA	TAA + NA	TAA + VB2	TAA + VC	TAA + NA,VB2,VC
MDA	6.43 ± 0.23	11.78 ± 0.45^a^	8.59 ± 0.42^a,b^	9.04 ± 0.47^a,b^	8.16 ± 0.32^a,b^	7.35 ± 0.16^a,b,c^
GSH	32.78 ± 1.06	19.68 ± .0.30^a^	23.79 ± 0.51^a,b^	23.55 ± 1.02^a,b^	25.82 ± 0.54^a,b^	29.48 ± 0.49^b,c^
Catalase	503.17 ± 30.00	266.84 ± 22.57^a^	400.46 ± 20.33^a,b^	370.16 ± 12.93^a,b^	395.00 ± 18.71^a,b^	468.52 ± 8.75^b,c^
SOD	275.43 ± 8.41	131.73 ± 6.98^a^	200.17 ± 11.07^a,b^	180.00 ± 4.51^a,b^	188.72 ± 9.12^a,b^	249.84 ± 8.26^b,c^
NO	65.78 ± 3.18	127.34 ± 6.38^a^	80.67 ± 3.74^a,b^	93.08 ± 4.11^a,b^	88.71 ± 3.53^a,b^	74.96 ± 2.56^b^
H.Prol	112.47 ± 6.32	763.71 ± 21.49^a^	426.85 ± 10.65^a,b^	512.67 ± 17.53^a,b^	459.27 ± 13.89^a,b^	342.79 ± 8.29^a,b,c^
TNF-α	12.42 ± 0.25	76.28 ± 1.03^a^	44.90 ± 0.56^a,b^	60.85 ± 0.52^a,b^	53.67 ± 0.36^a,b^	36.42 ± 0.11^a,b,c^

^a^Significant difference from control

^b^Significant difference from TAA group

^c^Significant difference from TAA + NA, TAA + VB2, TAA + VC

### Histopathological investigation of the liver

#### Control hepatic tissue

**Fig. 1:**
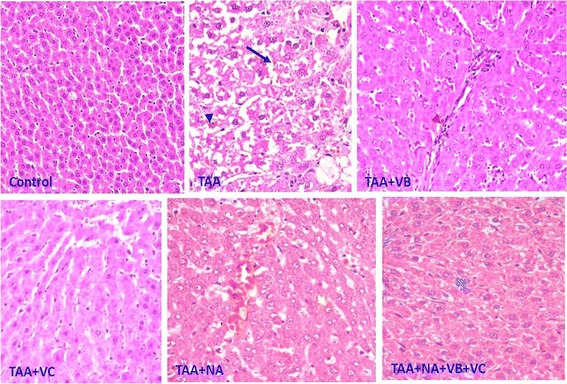
Representative photos of the hepatic tissues from the control and the various treated groups. Arrow: Dilated sinusoids; Black arrow head: Vacuolated hepatocytes; Red arrow head: Inflammatory cell infiltration; Yellow arrow head: Vesiculated nucleus; Blue arrow head: Normal nucleus

Figure 1 shows the typical structure of the hepatic tissues with normal polygonal hepatocytes and normal blood sinusoids.

#### The effect of TAA

Histopathological examination of hepatic sections from TAA rats revealed the presence of vacuolated hepatocytes with degenerated nuclei and irregular and damaged cell membranes. Disturbed and dilated blood sinusoids were also clearly observed along with fibroblasts distributed extensively through the hepatic tissue of this group. Moreover, mitotic figures were also noted in some hepatic sections (Fig. 1).

#### The effect of combining TAA with treatment with VB

The sections from this group showed an increased number of irregular hepatocytes with degenerated nuclei. Disturbed and narrowed blood sinusoids, frequently infiltrated with inflammatory cells, were also a prominent feature in the liver sections of these rats, along with fibroblasts extensively distributed through the hepatic tissue (Fig. 1).

#### The effect of combining TAA with treatment with VC

The general structure of the hepatic tissue in this group was markedly improved. Hepatocytes with faint cytoplasm but with healthy nuclei were arranged in hepatic cords creating narrow blood sinusoids (Fig. 1).

#### The effect of combining TAA with treatment with NA

Histological examination of different sections from rats of this group revealed that there was a marked improvement in the distribution of hepatocytes relative to the hepatic blood sinusoids. On the other hand, hepatocytes appeared with vesiculated nuclei showing partially degenerated chromatin materials.

#### The effect of combining TAA with treatment with VC and VB

Although there was an apparent improvement in the general hepatic architecture, many pathological signs could still be observed in the sections of this group. Some hepatocytes appeared vacuolated, and the blood sinusoids were narrow and disturbed in comparison with those in the control sections (Fig. 1).

## Discussion

Liver disease has been one of the traditionally major health issues in Egypt for a long time. The primary causes of liver disease are schistosomiasis (from the 1950s until the 1980s) and hepatitis C virus (1990s). Here, we used TAA to induce an experimental model of liver damage to mimic the pathophysiological symptoms of acute human liver diseases. TAA causes hepatic fibrosis through liver cell membrane injury, which in turn, causes a marked increase in enzymes levels. In the present study, TAA caused a significant increase in ALT, AST, ALT, LDH and bilirubin levels versus to the control the ones. Elevation of the activity of liver enzymes after TAA administration indicates cellular leakage, loss of structural and functional integrity of the liver [31].

We have previously reported several natural products with antioxidant and anti-inflammatory properties in vivo studies with promising results [32–35]. The present investigation has shown that rats treated with TAA have increased hepatic Nitric oxide (NO) and plasma TNF-α. Although NO is an important signaling molecule; yet its excess production results in the formation of peroxynitrite which is a very invasive cellular oxidant [36]. It is interesting, therefore to report that both these parameters were effectively countered in the animals administered with NA, VB2 or VC in the present study.

Lots of literature on anti-oxidative therapy and antioxidants have been proposed to prevent and treat liver diseases [37] highlighting the curative properties of antioxidants in such ailments. Specifically, treatment of TAA rats with NA improved the activities of liver enzymes and the histopathological structure of the liver. Earlier, we have reported that NA exerts a hepatoprotective effect against a high-fat ethanol diet and acetaminophen-induced acute liver injury [38]. Besides, it is documented that NA benefits both dams and pups; hence it merits evaluation for preventing or treating Preeclampsia complication in human pregnancies [39]. Furthermore, NA has been effective in ameliorating many oxidative stress-induced pathological conditions [40].

Similarly, VC also normalized the increased activity of liver enzymes in the plasma samples confirming its hepatoprotective effects in the present investigation. Studies have shown that this vitamin has hepato-protective properties due to its antioxidative properties, that gets better in synergy with other agents. The co-administration of ascorbic acid with alpha-tocopherol acetate and sodium selenate ameliorated ethanol-induced liver damage in rats [41]. The vitamin is believed to normalize the liver function parameters like ALT, AST, LDH, ALP, some endogenous antioxidants as well as blood hydroperoxide and malondialdehyde in the livers of carbon tetrachloride intoxicated rats [42, 43]. Besides, it has shown restoration and reconstitution of the polyfunctional, long-lived T-cells in the diabetic rats [44]. Furthermore, the vitamin is the most important hydrophilic antioxidant with high efficiency of free radical scavenging in vivo [45]. However, the mechanism involved appears to be ascorbyl radical reacting with other free radicals to stop their propagation [46].

It is documented that VB2 significantly improves serum and the histological parameters of hepatocellular damage and neutrophil infiltration following liver ischemia in mice [17]. Recently, many studies have also demonstrated that irradiated riboflavin protects the cisplatin-induced oxidative stress-mediated hepatotoxicity and nephrotoxicity in rodent based studies [47–50]. Furthermore, VB2 and UV light together were able to reduce the titer of MERS-CoV in human plasma products to a non-detecting level suggesting that the combination may lower the risk of transfusion transmission of MERS-CoV [51]. In this study treatment of rats with the three vitamins in combination led to a more pronounced alleviation of the effects of the liver injury induced by TAA. We have previously found that folic acid and melatonin as a combination was superior to using them individually regarding recovering hepatic function and structure [51]. In the present data, TAA administration resulted in a significant reduction in the plasma levels of a total protein that might be indicative of injury in the hepatocytes and excessive destruction of proteins including antioxidant enzymes and cellular reducing powers including SH-protein bond production or alterations in RNA sequences in the target tissues. An increase in ALP activity and bilirubin level after TAA treatment reflects the pathological change in the biliary flow in the animals. Likewise, their suppression in the rats treated with NA, VB2, and VC as combination suggests possible stabilization of the biliary dysfunction. Furthermore, TAA also enhanced the extent of lipid peroxidation, liver fibrosis and cirrhosis accompanied by the morphological and many other biochemical alterations resembling that of the human disease [52, 53]. Therefore, it is logical to say that TAA caused the cellular damage by inhibiting the activity of the antioxidant enzymes in the present study [54]. The combination of three vitamins significantly decreased liver hydroxyproline content in TAA-treated rats, which may be due to a decline in the accumulation of fibrotic tissue in the liver. Our findings are in accord with many earlier studies emphasizing the role of oxidative stress in the mechanisms of liver fibrosis and cirrhosis [55]. The antioxidant enzymes (SOD and CAT) as well as reducing power GSH were found significantly improved in the liver homogenates of the rats treated with TAA followed by the three-vitamins combination as compared to the control. The improvement of all these parameters seems to restore the structural and functional integrity of the target organs in the animals [56].

Hyperlipidaemia is a vascular risk factor associated with atherosclerosis, coronary artery diseases, cerebral vascular disease and peripheral disease. The present investigation showed that the three vitamin combination inhibits the increase in cholesterol, triglycerides and LDL levels in TAA treated rats. However, the combination increased the HDL level. Our study showed that TAA toxicity stimulated hypercholesterolemia with hypertriglyceridemia. VC may hinder the development of atherosclerosis and prevent the onset of acute coronary events by several molecular mechanisms. The vitamin also helps in maintaining arterial wall integrity that can alter cholesterol metabolism by modulating the conversion of cholesterol into bile acids, while it affects plasma triglyceride levels via modulation by lipoprotein lipase activity [57]. The vitamin has also been shown to protect HDL cholesterol from lipid oxidation. It, therefore, allows for reverse cholesterol transport involving the removal of unesterified cholesterol from extrahepatic cell membranes into esterified ones via lecithin: cholesterol acyltransferase [58]. Apart from these, another vitamin taken under the present study, nicotinamide has been reported to decrease serum cholesterol, triglycerides and free fatty acids in the fasting rat [59]. Moreover, NA induced up-regulation of tumor necrosis factor-α (TNF-α) transcription and interleukin-6 (IL-6). Both the cytokines are with lipolytic properties, thereby increasing free fatty acid release [60].

**Fig. 2:**
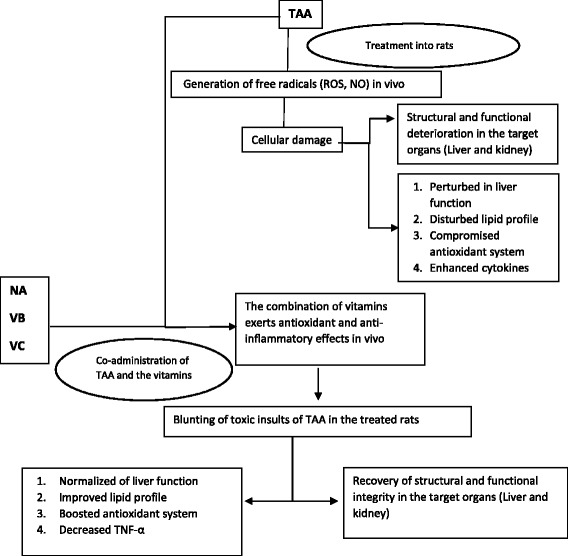
Showing putative mechanism of TAA-toxicity amelioration by the proposed combination of the vitamins

All the three vitamins have an excellent antioxidant and anti-inflammatory properties. Upon their co-administration in TAA pre-treated rodents, they decreased the oxidative stress that further improved cellular structure and functionality. The diminished liver function markers and cholesterol, LDL and TNF-α concomitant with enhanced activity of antioxidant enzymes (SOD and CAT) and replenishment of the cellular reducing power (GSH) are vivid indicators of recovery of tissue damage. The improvement in all these parameters indicates that the cellular, molecular and structural damage incurred by TAA were alleviated by the combination of the vitamins. As each vitamin has a different degree of antioxidant and anti-inflammatory properties, their respective combination gives a different level of outcome after the treatment. However, when all the vitamins were administered together, they showed a synergistic ameliorative effect against the TAA-induced toxic insults in vivo (Fig. 2).

## Conclusions

The present study entails the combination of NA, VB2, and VC has stronger antioxidant and anti-inflammatory power as compared to the individual potential of the vitamins against TAA-induced toxicity. The three vitamins exert the protective effects through their antioxidant, antifibrosis and anti-inflammatory properties that consequently restore the structure and functionality of the target organs. Hence, anti-oxidative therapy mainly comprising of natural antioxidants represents a reasonable therapeutic approach for the prevention and treatment of oxidative stress-mediated liver diseases.

## Abbreviations

CAT: Catalase
GSH: Glutathione
HDL: High density lipoprotein
LDL: Low density lipoprotein
MDA: Malondialdehyde
NA: Nicotinamide
NO: Nitric oxide
S.E.M.: Tumour necrosis factor-alpha (TNF-α), Standard error of mean
SOD: Superoxide dismutase
TAA: Thioacetamide
VB2: Vitamin B2
VC: Vitamin C
